# A sustainable approach for the extraction of cholesterol-lowering compounds from an olive by-product based on CO_2_-expanded ethyl acetate

**DOI:** 10.1007/s00216-019-01970-4

**Published:** 2019-07-06

**Authors:** Romy Vásquez-Villanueva, Merichel Plaza, María Concepción García, Charlotta Turner, María Luisa Marina

**Affiliations:** 1 Departamento de Química Analítica, Química Física e Ingeniería Química, Universidad de Alcalá, Ctra. Madrid-Barcelona Km. 33.600, 28871 Alcalá de Henares, Madrid Spain; 2 Department of Chemistry, Centre for Analysis and Synthesis, Lund University, P.O. Box 124, 22100 Lund, Sweden; 3 Instituto de Investigación Química Andrés M. del Río, Universidad de Alcalá, Ctra. Madrid-Barcelona Km. 33.600, 28871 Alcalá de Henares, Madrid Spain

**Keywords:** Bio-based solvents, Cholesterol-lowering capacity, CO_2_-expanded liquid, Hansen solubility parameters, Olive seed, Phenolic compounds, Phytosterols

## Abstract

Olive (*Olea europaea*) processing results in large amounts of by-products that contain valuable molecules such as phenolic compounds and phytosterols. These molecules have demonstrated to reduce blood cholesterol levels. This work proposes the development of a method to obtain simultaneously phenolic compounds and phytosterols from the olive stone using CO_2_-expanded liquid extraction. Hansen solubility parameters were employed for the theoretical prediction of the most suitable bio-based solvent to extract target compounds. The Box–Behnken experimental design was employed to select the optimal conditions of pressure (8–25 MPa), the molar fraction of CO_2_ in ethyl acetate (0.15–0.55), and the temperature (40–80 °C). Extracts showing the highest and the lowest reductions of micellar cholesterol solubility capacity were analyzed by gas chromatography coupled to mass spectrometry to find out the compounds responsible for this activity. Different phenolic compounds, free fatty acids, and phytosterols were identified in the extracts. β-Sitosterol and, especially, tyrosol and hydroxytyrosol were the compounds that primarily contributed to the reduction of micellar cholesterol solubility capacity.

**Electronic supplementary material:**

The online version of this article (10.1007/s00216-019-01970-4) contains supplementary material, which is available to authorized users.

## Introduction

There is an increasing interest for obtaining bioactive compounds from natural sources for the elaboration of functional foods, nutraceuticals, pharmaceuticals, and cosmeceuticals. On the other hand, food industry by-products constitute an important problem from environmental and economic points of view. However, some food industry by-products are cheap and natural sources of compounds with valuable functional properties that can be of interest for the food industry itself and for the pharmaceutical and cosmetic industries [1, 2].

Olive processing generates a large amount of by-products which are made up basically of leaves, wood, peel, and stones which tend to be underused and undervalued [3]. Several studies reported the potential reuse of olive stones for animal feeding, composting, and biofuel and energy production because of their high protein and oil content [4–6]. Additionally, recent researches proposed a new strategy for exploiting olive stones. This strategy is based on the extraction of olive stone proteins followed by their hydrolysis to obtain valuable peptides with antioxidant, antihypertensive, antitumoral, and hypocholesterolemic properties [7–9]. Another important part of the olive seed is its oil fraction. Olive seed oil presents an interesting nutritional profile due to the presence of fatty acids, vitamins, carotenoids, phytosterols, phenolic compounds, and other compounds, which have a significant role in aroma and in the chemical properties of olive [10]. The consumption of these compounds is linked to the prevention and/or reduction of the metabolic syndrome risk (which includes obesity, hyperglycemia, dyslipidemia, and hypertension diseases) [11]. Olive seed oil has been extracted using conventional methodologies requiring high amounts of polluting organic solvents [12]. Therefore, the development of sustainable extraction methods using nonpolluting solvents is highly desirable. The selection of a suitable bio-based solvent to simultaneously extract phenolic compounds and phytosterols constitutes a great challenge since these compounds present very different polarities. Indeed, phytosterols possess mainly nonpolar behavior, while phenolic compounds possess a higher solubility in intermediate polarity solvents, rather than polar or nonpolar solvents. Moreover, the intermolecular forces between phenolic compounds and solvents depend on the number of hydroxyl groups [13].

CO_2_-expanded liquid (CXL) extraction is an advanced extraction technique in which a solvent is pressurized with CO_2_ and is volumetrically expanded [14, 15]. The addition of a compressed gas in an organic solvent could improve the extraction process. Indeed, solvent expansion modifies both solvent characteristics and physical properties. It reduces the relative permittivity as well as the hydrogen-bonding abilities of the solvent, it improves mass transfer through the reduction of solvent viscosity, and thus, it increases solute diffusivity and decreases interfacial tension. In addition, the use of CXLs can lead to a general reduction in the use of solvents even “up to 80%” [15]. These improvements will depend on the temperature, pressure, and proportion of CO_2_ in the organic solvent. The use of CXLs in extraction processes has not been studied in depth. In fact, there are few works in the literature that describe the use of CXLs based on methanol for the extraction of lipids [16] and based on ethanol for the extraction of astaxanthin [17], carotenoids [18], γ-linolenic acid [19], and monoterpenes [20]. These works have revealed the great potential of the extraction with CXLs as a green extraction technology.

A sustainable extraction process also requires the use of nonpolluting solvents. “Green” solvents should be inexpensive, easily removed, nontoxic, environmentally benign, biodegradable, and obtained from renewable feedstock [21]. Bio-based solvents are promising “green” solvents since they are derived from renewable sources, generally agricultural crops or residual organic matter considered as waste. For instance, during the fermentation of waste sugars, it is possible to obtain ethanol, ethyl acetate, or ethyl lactate; other solvents can be obtained from chemical conversion of lignocellulosic waste such as 2-methyltetrahydrofuran or methanol, or from cooking oil waste such as glycerol, or from citrus peel such as the terpene D-limonene [22]. They constitute a remarkable alternative to petroleum-based solvents [22]. Moreover, the use of theoretical approaches for the selection of solvents, like Hansen solubility parameters (HSP), enables to reduce the number of experiments, avoiding solvent waste generation and reducing time [23]. This approach is based on a group-contribution method used to evaluate the miscibility among substances and solvents assessing the affinity among them. Despite HSP being employed for the extraction of some bioactive compounds [20, 24–27], they have never been used for the simultaneous extraction of compounds with such a different polarity as phytosterols and phenolic compounds.

Therefore, the aim of the present work was the development of a sustainable and efficient extraction method to recover compounds with cholesterol-lowering properties (i.e., phenolic compounds and phytosterols) from olive seeds. Theoretical predictive assessment of HSP was employed to select the suitable bio-based solvent to extract phenolic compounds and phytosterols. The Box–Behnken design was used to select optimal conditions to obtain extracts with the highest in vitro cholesterol-lowering capacity from olive seeds using CXL technology. Gas chromatography (GC) coupled to mass spectrometry (MS) was employed to identify those compounds responsible for this activity.

## Materials and methods

### Materials

Ethyl acetate was from Fisher Scientific UK (Bishop, Meadow Road, Loughborough). Ultrapure CO_2_ in cylinder containers with a dip tube was purchased from Air Products (Amsterdam, Netherlands). Sodium dihydrogen phosphate was from Merck (Darmstadt, Germany) and methanol (MeOH) was from Scharlau Chemie (Barcelona, Spain). Cholesterol oxidase kit was purchased from BioAssay Systems (Hayward, CA USA). Taurocholic acid, oleic acid, phosphatidylcholine, *N,O*-bis(trimethylsilyl)trifluoroacetamide with trimethylchlorosilane (BSTFA + 0.1% TMCS), tyrosol, hydroxytyrosol, β-sitosterol, and *n*-alkane standard solution C_8_–C_40_ were purchased in Sigma (St. Louis, MO, USA). The olives of ‘Manzanilla’ variety were kindly donated by FAROLIVA S.L. Company (Murcia, Spain).

### Olive seed sample pretreatment

Olives were manually pitted and seeds inside olive stones were extracted with a nutcracker. Olive seeds were ground using a grinder and dried for 48 h using a freeze-dryer (Hetosicc, Heto Birkerød Denmark). Dried olive seed samples were stored at − 80 °C until use.

### Solvent selection by Hansen solubility parameters

HSP were employed to estimate the solubility of phenolic compounds and phytosterols in bio-based solvents. This estimation is based on the prediction of the kind of interactions established between target compounds and solvents. Three different molecular interactions were taken into account: dispersive interaction (*δ*_D_), that is related to Van der Waals forces and other molecular forces; polar interaction (*δ*_P_), that is related to dipole moment; and hydrogen-bonding interactions (*δ*_H_). A three-dimensional plot of HSP (*δ*_D_, *δ*_P_, *δ*_H_) results in a “solubility” sphere that enables to select a suitable solvent to extract a target compound. “Good solvents” will present HSP similar to those observed for target compounds, and solubility spheres will be close or even overlapped. The radius of this sphere is called “interaction radius” (*R*_0_). The distance between the sphere centers corresponding to a solute and a solvent is *R*_a_. The ratio *R*_a_/*R*_0_, called relative energy difference (RED), yields valuable information on the interaction between a target compound and a solvent. A RED value ≤ 1.0 indicates a high affinity of the target compound for the solvent, while a RED value > 1.0 indicates a low affinity [28].

The software Hansen Solubility Parameters in Practice (HSPiP) from the official Hansen solubility parameter site [https://www.hansen-solubility.com] was used to predict the HSP. Prediction is based on physical properties and solubility parameters of target compounds. In the case of phenolic compounds and phytosterols, this information was not available and the Yamamoto-molecular break method using its simplified molecular input line entry syntax (SMILES) was employed for its estimation.

Since HSP of an organic solvent is modified by the presence of a compressed gas, corrected HSP were calculated by the approach proposed by Williams et al. [29]

1 $$ {\delta}_{\mathrm{D}}={\delta}_{\mathrm{D},\mathrm{ref}}{\left(\frac{V_{\mathrm{ref}}}{V}\right)}^{1.25} $$

2 $$ {\delta}_{\mathrm{P}}={\delta}_{\mathrm{P},\mathrm{ref}}{\left(\frac{V_{\mathrm{ref}}}{V}\right)}^{0.5} $$

3 $$ {\delta}_{\mathrm{H}}={\delta}_{\mathrm{H},\mathrm{ref}}{\left[{\mathrm{e}}^{\left(-1.32\times {10}^{-3}\ \Big({T}_{\mathrm{ref}}-T\right)-{\left(\frac{V_{\mathrm{ref}}}{V}\right)}^{0.5}}\right]}^{-1} $$

where *δ*_D_, *δ*_P_, and *δ*_H_ are the corrected HSP considering the temperature effect. *V* is the molar volume at the desired temperature (*T*) and pressure. All parameters with “ref” subscript (*δ*_D,ref_, *δ*_P,ref_, and *δ*_H,ref_, and *V*_ref_) are referred at room temperature (*T*_ref_ = 25 °C) and atmospheric pressure.

HSP corresponding to a mixture of solvents were determined considering this equation [28],

4 $$ {\delta}_{\mathrm{mixture}}=\sum {\gamma}_{\mathrm{i}}{\delta}_{\mathrm{T},\mathrm{i}} $$

where *γ*_i_ is the composition of every solvent in molar fraction (in percentage), and *δ*_T, i_ is the total HSP of solvent.

### Design of the experiments

To optimize the influence of the temperature, pressure, and CO_2_ molar fraction (X_CO2_) on the extraction of cholesterol-lowering compounds such as phenolic compounds and phytosterols, a response surface methodology was employed. The Box–Behnken design (MODDE 10.1, Sartorius Stedim Biotech, Malmö, Sweden) was selected since it is a second-order design based on three levels. The extraction pressure ranged from 8 to 25 MPa, temperature from 40 to 80 °C, and X_CO2_ was from 0.15 to 0.55. Five different responses were determined in the extracts obtained under selected conditions: extraction yield (% dry extract weight obtained/initial dry sample weight, *w*/*w*), in vitro reduction of micellar cholesterol solubility capacity assay (%) (cholesterol-lowering capacity), and phenolic compounds, phytosterol, and free fatty acid contents (expressed as total peak area). Analysis of variance (ANOVA) was applied to evaluate the adequacy of fitted models established between temperature, pressure, and X_CO2_ and the different responses.

### CO_2_-expanded ethyl acetate extraction

CXL extraction of freeze-dried olive seeds was performed using a MV-10 ASFE system (Waters Technologies, Milford, MA, USA) controlled by a ChromScope™ software (Waters Technologies, Milford, MA, USA). The system consisted of a dual piston pump for delivering CO_2_ and co-solvents connected with a T-junction, an oven, an automated back pressure regulator (BPR), a transfer line heated with a heat exchanger, a make-up solvent pump, and a fraction collecting module. For each experiment, the extraction cell was filled with 500 mg of freeze-dried olive seed. CO_2_ and ethyl acetate were pumped at a constant flow rate (2.0 mL/min) and then passed through a 200-cm preheating coil inside the oven. The pressure was controlled by the BPR. Ethyl acetate was introduced at a flow rate of 0.3 mL/min after BPR in order to pick up all the analytes in the collecting vessel after the expansion of CO_2_. The extract was collected in a 25-mL glass bottle. According to a previous kinetic study, extraction time was set at 10 min to ensure high extraction yield [20]. Between extractions, the system was flushed with CO_2_/ethyl acetate mixture under previous extracting conditions to avoid carryover. Extracts were stored at − 80 °C until analysis.

### In vitro assay for the evaluation of the reduction of micellar cholesterol solubility (RMCS) capacity

Micelles were synthesized according to the method reported by Zhang et al. [30] with slight modifications. A solution consisting of 0.5 mM cholesterol, 1 mM oleic acid, and 2.4 mM phosphatidylcholine in MeOH was prepared and dried at room temperature overnight; 15 mM phosphate buffer (pH 7.4) containing 6.6 mM taurocholate salt and 132 mM NaCl was added to the lipid blend. To form the micelles, the mixture was sonicated for 1 min at 95% of amplitude by using a high-intensity focusing ultrasound probe (Sonic Vibra Cell, CVX 130, Hartford, CT, USA) followed by its incubation overnight at 37 °C in a Thermomixer Compact (Eppendorf, Hamburg, Germany). For the assay, 150 μL of 10 mg/mL of sample was added to 50 μL of the micelle solution, sonicated for 1 min at 95% of amplitude, and incubated for 2 h at 37 °C. The mixture was centrifuged for 10 min at 6000×*g*. The supernatant was collected for the determination of the cholesterol remaining in micelles using a cholesterol kit, which was based on the cholesterol oxidase method. Cholesterol in micelle was calculated by interpolation in a calibration curve obtained using cholesterol as standard. The reduction in the micellar solubility of cholesterol was calculated using the following equation:

5 $$ \mathrm{RMCS}\ \left(\%\right)=\left(\frac{C_0-{C}_{\mathrm{i}}}{{\mathrm{C}}_0}\right)\times 100 $$

where *C*_0_ is the initial concentration of cholesterol in micelles and *C*_i_ is the concentration of cholesterol in micelles when adding the extract.

### Gas chromatography analysis of extractable fraction from olive seeds

#### Preparation of trimethylsilyl ether (TMS)-derivative compounds

CXL seed extracts were evaporated under a gentle nitrogen stream before derivatization. Afterwards, 50 μL of BSTFA (with 1% TMCS) was added to the dried residue for silylation. The mixture was vortexed for 15 min and heated at 80 °C for 1 h. After cooling, 1 μL of the sample was injected into the GC system.

#### Gas chromatography–mass spectrometry analysis

Analyses were carried out on an Agilent GC system 7890B from (Agilent Technologies, Palo Alto, CA, USA) with a ZB-5HT inferno capillary column (5% phenyl, 95% dimethyl-polysiloxane high-temperature phase, 30 m × 0.25 mm I.D., 0.25 μm film thickness) from Phenomenex Inc. The system was controlled by means of Agilent MSD ChemStation software. The chromatographic separation was based on a previous method with some modifications [31]. Optimal separation conditions were as follows: 150 °C for 2 min, then increased to 350 °C at a rate of 5 °C/min. The injector was heated to 270 °C in the split mode (ratio 1:20). Helium was used as carrier gas (7 psi). MS conditions were energy 70 eV and full scan mode from *m*/*z* 50 to 700. Identification of compounds was carried out by mass spectra analysis using NIST 05 mass spectral library, by comparison with data found in the literature, and by comparison with standards, when available. In addition, their linear retention indices (RIs) were calculated to identify compounds more accurately. To make this, a mixture of hydrocarbons (*n*-octane to *n-*tetracontane) dissolved in *n*-hexane was used. Moreover, the relative and normalized area (%) and the total peak area of every compound were also estimated. The normalized area was calculated as follows:

6 $$ \%A=\frac{A_{\mathrm{i}}}{A_{\mathrm{total}}}\times 100 $$

where *A*_i_ is the peak area of each compound and *A*_total_ is the total area resulted in the chromatogram.

## Results and discussion

This work proposes the valorization of olive seeds based on the extraction of phytosterols and phenolic compounds. These compound classes have demonstrated cholesterol-lowering properties, but they have never been simultaneously extracted from olive seeds. For that purpose, a suitable bio-based solvent will be employed.

### Selection of bio-based solvent using Hansen solubility parameters

The selection of the bio-based solvent was carried out considering the main phytosterol (β-sitosterol) and phenolic compounds (tyrosol and hydroxytyrosol) in the olive seed. Four different bio-based solvents covering a broad range of polarities were employed: ethanol, D-limonene, ethyl lactate, and ethyl acetate. Table 1 summarizes the HSP estimation for these compounds and tested solvents. Since the information on physical properties and solubility of phenolic compounds and phytosterols was not available in the HSPiP software, determination of HSP was performed considering the properties estimated from their molecular structure (SMILES) using the Yamamoto-molecular break method.

**Table 1:** Hansen solubility parameters of target compounds (β-sitosterol, tyrosol, and hydroxytyrosol) and bio-based solvents (ethanol, D-limonene, ethyl lactate, and ethyl acetate) (at room temperature and 1 atm)

Compound	SMILES	Molar volume (cm^3^ mol^−1^)	*δ*_D_ (MPa^½)^	*δ*_P_ (MPa^½^)	*δ*_H_ (MPa^½^)	*δ*_Total_ (MPa^½^)
β-Sitosterol	CCC(CCC(C)C1CCC2C1(CCC3C2CC=C4C3(CCC(C4)O)C)C)C(C)C	436.5	17.1	1.9	3.0	17.5
Tyrosol	C1=CC(=CC=C1CCO)O	122.3	19.3	8.1	16.8	26.8
Hydroxytyrosol	C1=CC(=C(C=C1CCO)O)O	124.6	19.7	9.1	19.1	28.9
Ethanol	CCO	58.6	15.8	8.8	19.4	26.5
D-limonene	CC1=CCC(CC1)C(=C)C	162.9	17.2	1.8	4.3	17.8
Ethyl lactate	CCOC(=O)C(C)O	115.0	16.0	7.6	12.5	21.7
Ethyl acetate	O=C(OCC)C	98.6	15.8	5.3	7.2	18.2

**Fig. 1:**
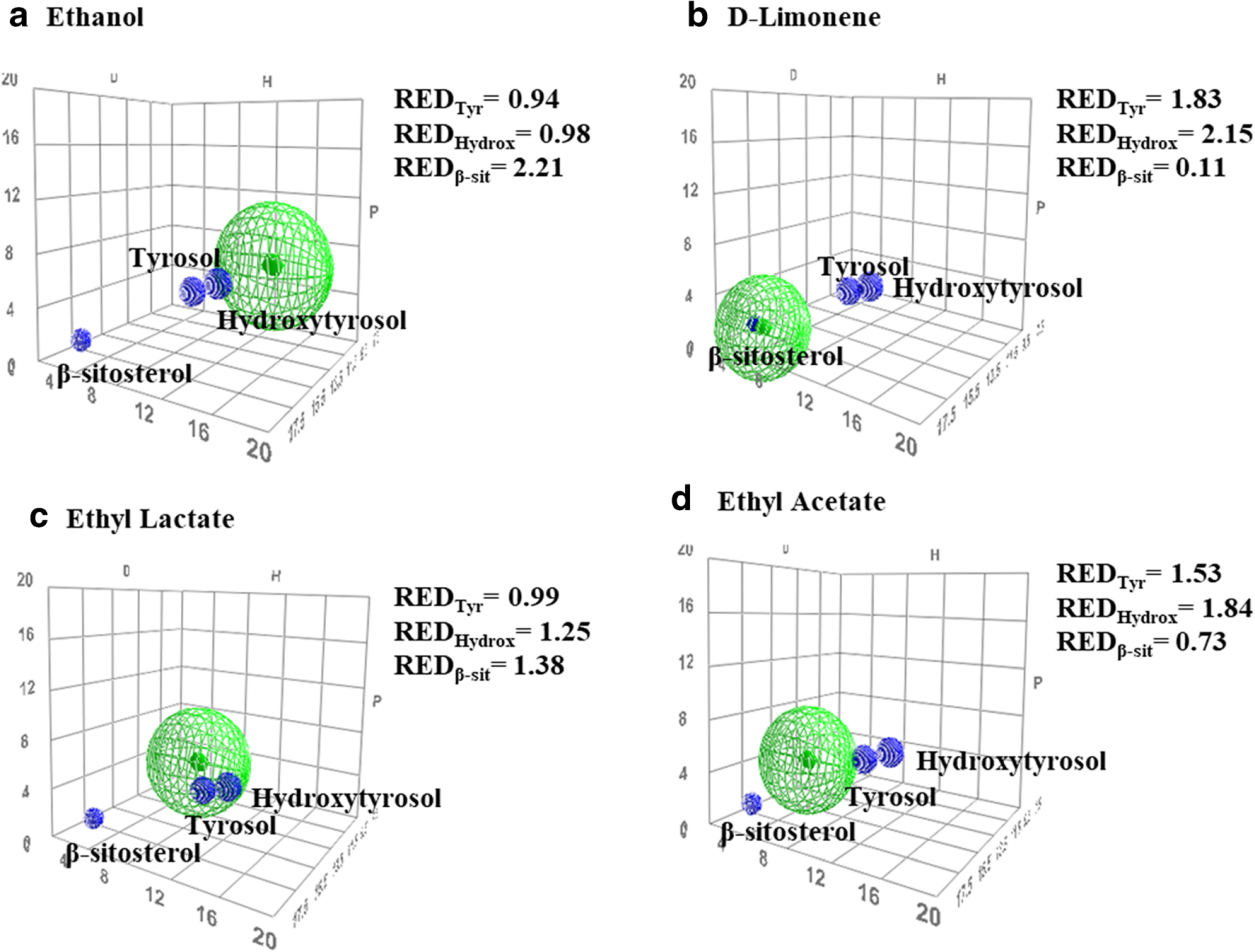
Three-dimensional representation of Hansen solubility spheres corresponding to β-sitosterol, tyrosol, and hydroxytyrosol (blue dots) and to solvents (green spheres) ethanol (**a**), D-limonene (**b**), ethyl lactate (**c**), and ethyl acetate (**d**). RED values are provided in every figure

As expected, β-sitosterol (*δ*_P_ = 1.9 MPa^1/2^) and phenolic compounds (*δ*_P_ = 8.1 MPa^1/2^ and *δ*_P_ = 9.1 MPa^1/2^ for tyrosol and hydroxytyrosol, respectively) showed very different polarities which limited the selection of a suitable bio-based solvent. HSP can be represented in a three-dimensional space enabling a visual approximation of the target compounds and the bio-based solvents. Figure 1 shows the three-dimensional HSP sphere for all evaluated compounds (phenolic compounds and phytosterols) and the interaction with the different studied bio-based solvents. Blue dots correspond to the target compounds, β-sitosterol, tyrosol, and hydroxytyrosol, while the green spheres represent the pure bio-based solvents, ethanol, D-limonene, ethyl lactate, and ethyl acetate. Good solvents are those whose green sphere contains or is close to the blue dots of target compounds, while when blue dots are far from the green sphere, it means that target compounds are not well dissolved in the specific solvent. This figure also shows the RED values for the interaction between every compound and the pure bio-based solvent. As it can be observed in Fig. 1a, phenolic compounds were close to pure ethanol (RED < 1), but β-sitosterol was far from the green sphere (RED > 1); thus, it was expected that ethanol can dissolve phenolic compounds but not β-sitosterol. On the contrary, D-limonene seemed to be a suitable solvent for the solubilization of β-sitosterol (RED < 1), but it was not useful for the solubilization of phenolic compounds (RED > 1) (see Fig. 1b). Finally, ethyl lactate and ethyl acetate seemed to offer a commitment between the solubility of phytosterols and phenolic compounds. In the case of ethyl lactate, phenolic compounds were close to the solvent, showing RED values of 0.99 and 1.25; meanwhile, β-sitosterol likely had a lower solubility, presenting a RED value of 1.38 (Fig. 1c). For ethyl acetate, β-sitosterol exhibited a RED value of 0.73, while tyrosol and hydroxytyrosol displayed RED values of 1.53 and 1.84, respectively (Fig. 1d).

The boiling point of ethyl acetate (77.1 °C) is lower than that of ethyl lactate (154 °C); thus, ethyl acetate is easily evaporated and it has been successfully applied for the extraction of phenolic compounds from olive residues [32]. It was the bio-based solvent finally selected. In fact, ethyl acetate was considered as an efficient solvent for the recovering of low and medium molecular weight phenolic compounds [32, 33].

### Optimization of the extraction of cholesterol-lowering compounds employing CO_2_-expanded ethyl acetate

Once ethyl acetate was selected to extract phytosterols and phenolic compounds, the effect of the addition of CO_2_ was evaluated. For this purpose, the influence of the temperature (40–80 °C), pressure (8–25 MPa), and molar fraction of CO_2_ in ethyl acetate (X_CO2_) (0.15–0.55) on two response variables (extraction yield and in vitro RMCS capacity) was evaluated. The temperature, pressure, and X_CO2_ in ethyl acetate were chosen since they control the solubility in CXL extractions. The values investigated for these variables were established based on the phase diagrams of the binary systems (CO_2_–ethyl acetate) at different temperatures, estimated by the software GPEC (see Electronic supplementary material (ESM) Fig. S1) [34]. Selected ranges for temperature, pressure, and X_CO2_ guaranteed that binary mixtures of ethyl acetate and CO_2_ were in liquid state (a region where the liquid CO_2_ is miscible in ethyl acetate) [15]. A response surface methodology called the Box–Behnken design was employed to optimize the number of experiments required to study the influence of the three variables (pressure, temperature, and X_CO2_). Table 2 groups the 15 experiments established by the experimental design. Three of these experiments were replicated at the central point. Additionally, Table 2 also shows the extraction yields and RMCS of extracts obtained in the 15 experiments. In order to find out any correlation between these responses (extraction yield and RMCS) and the pressure, temperature, and X_CO2_ used in the extractions, a multiple linear regression model was applied. Table 3 shows the coefficients of the established multiple linear regression. The regression model could explain 78 and 93%, respectively, of the extraction yield and RMCS variability. Moreover, the standard error (expressed as relative standard deviation (RSD)) of the regression model was below 4.8. Additionally, ANOVA was employed to evaluate the adequacy of the regression model and results were also included in Table 3. Both regression models were considered adequate since the *p* value for the regression test was lower than 0.05 and the *p* value for the lack-of-fit test was higher than 0.05.

**Table 2:** Experimental design obtained by Box–Behnken and total oil (yield, % *w*/*w*), in vitro cholesterol-lowering capacity (RMCS, %), and phenolic compounds, phytosterol, and free fatty acid (FFA) peak areas (*A*) in the extracts obtained under the established conditions

Experiment number	Run	Pressure (MPa)	Temperature (°C)	Molar fraction CO_2_	Yield (% *w*/*w*)	RMCS (%)	*A*_phenolic compounds_ (× 10^7^)	*A*_Phytosterol_ (× 10^7^)	*A*_FFA_ (× 10^9^)
1	7	8.0	40	0.35	47.4	27.0	0.42	1.34	1.63
2	14	25.0	40	0.35	37.7	32.3	1.21	1.46	1.54
3	12	8.0	80	0.35	40.8	46.5	0.81	1.50	1.54
4	11	25.0	80	0.35	38.8	60.6	1.30	1.48	1.50
5	8	8.0	60	0.15	48.3	53.5	0.83	1.70	1.61
6	4	25.0	60	0.15	41.6	52.4	0.85	1.85	1.72
7	2	8.0	60	0.55	40.2	60.1	1.35	1.79	1.61
8	1	25.0	60	0.55	37.8	62.3	1.03	2.22	1.92
9	15	16.5	40	0.15	37.0	33.1	0.59	1.45	1.52
10	3	16.5	80	0.15	42.5	72.6	3.45	1.54	1.52
11	6	16.5	40	0.55	43.6	54.5	1.54	1.70	1.62
12	5	16.5	80	0.55	41.5	58.6	1.27	1.88	1.66
13	9	16.5	60	0.35	42.8	51.5	0.70	1.60	1.57
14	10	16.5	60	0.35	40.6	41.1	0.52	1.68	1.59
15	13	16.5	60	0.35	44.1	39.5	0.62	1.58	1.58

**Table 3:** Coefficients of the multiple linear regression models that best fitted the responses (yield, reduction of micellar cholesterol solubility (RMCS), phenolic compounds (PC), and phytosterol) with the extraction parameters (temperature, pressure, and X_CO2_) and analysis of variance (ANOVA)

Parameters	Yield	*p* value	RMCS	*p* value	PC	*p* value	Phytosterol	*p* value
Constant	43.0857	4.42372e-11	44.0333	9.51199e-07	− 0.215732	0.00811178	1.62	5.27076e-07
*P*	− 3.275	0.00493027	2.56251	0.174945	0.0699889	0.161739	0.0850002	0.0391249
*T*	− 1.8	0.0676772	11.425	0.000270002	0.125399	0.024595	0.0562502	0.12571
X_CO2_	− 0.225	0.798466	2.9875	0.121854	0.0348503	0.464759	0.13125	0.00782485
*P* ^2^	− 1.98572	0.150155	− 0.029164	0.991006			0.0362499	0.457877
*T* ^2^			− 2.40417	0.368067	0.148791	0.0560227	− 0.21125	0.00540606
X_CO2_^2^			13.0708	0.00121182	0.21511	0.0120844	0.23375	0.00351141
*P* × *T*	2.12499	0.115928					− 0.035	0.455781
*P* × X_CO2_	2.225	0.102117					0.07	0.167007
*T* × X_CO2_			− 8.85	0.00779712	− 0.21304	0.0105698	0.0224999	0.62562
*R* ^2^	0.78		0.93		0.818		0.94	
RSD	2.4		4.8		0.1284		0.08663	
*p* value (test of regression)	0.024		0.001		0.012		0.011	
*p* value (lack of fit)	0.152		0.845		0.164		0.215	

**Fig. 2:**
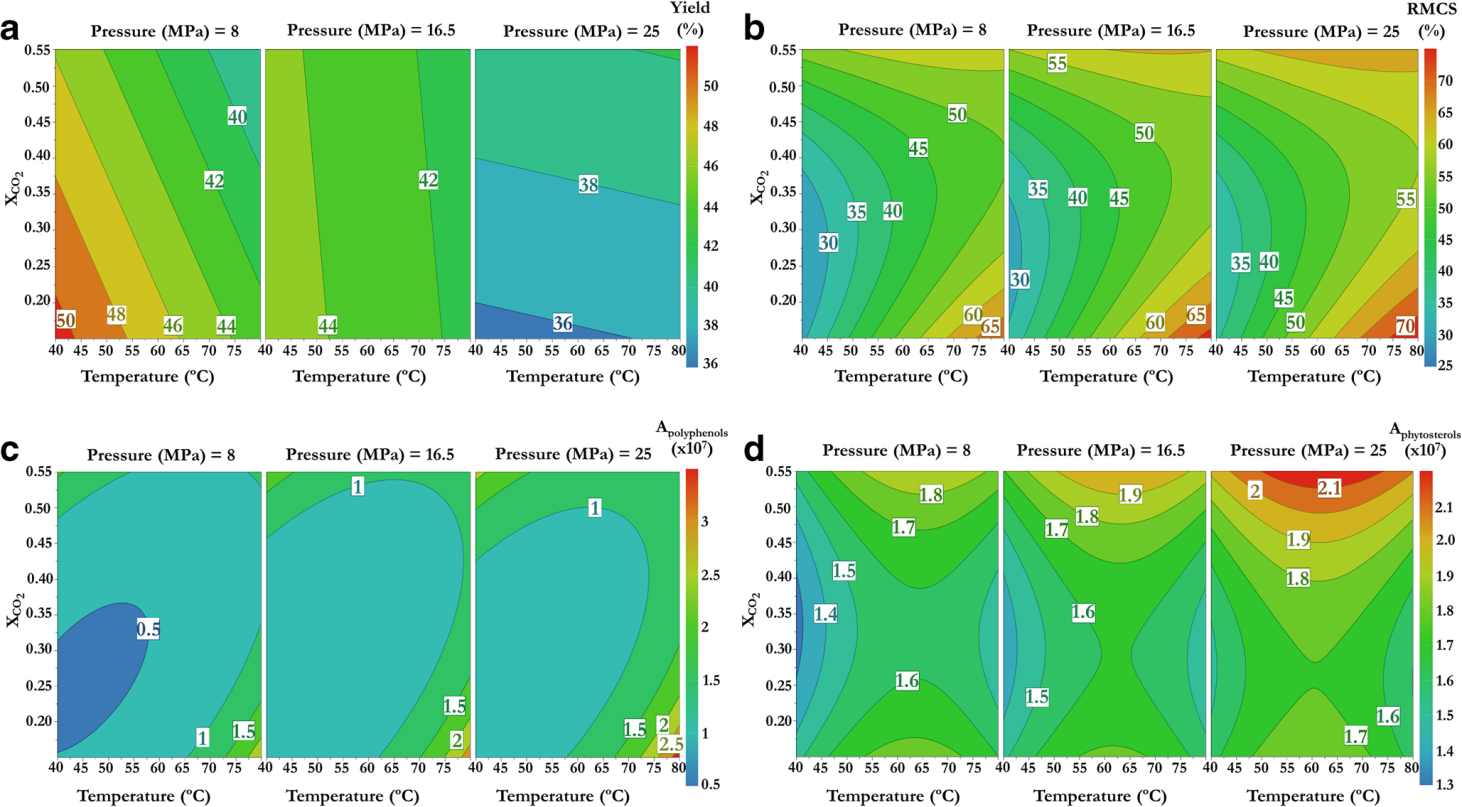
Contour plots showing the effect of pressure (MPa), temperature (°C), and CO_2_ molar fraction (X_CO2_) on the extraction yields, % *w*/*w* (**a**); on the reduction of micellar cholesterol solubility (RMCS), % (**b**); and on the phenolic compounds (**c**) and phytosterol (**d**) total peak areas

The effect of pressure, temperature, and X_CO2_ on the extraction yield and RMCS capacity is shown as contour plots in panels a and b of Fig. 2, respectively. According to Fig. 2a and data grouped in Table 2, the pressure was negatively correlated with the extraction yield. Moreover, a negative correlation of the extraction yield with temperature was also observed at pressures below 20 MPa, while temperature did not have any influence at higher pressures. Figure 2a also shows that the optimal conditions within the test range to obtain the highest extraction yields (*w*/*w*) from olive seeds were as follows: the lowest pressure (8 MPa), a temperature of 40 °C, and a X_CO2_ of 0.15. Under these conditions, the predicted extracted amount of solutes was 50.7 wt%.

On the other hand, the in vitro RMCS capacity of the extracts seemed to be positively correlated with temperature, especially at lower X_CO2_ (see Fig. 2b). Moreover, the optimal theoretical extraction conditions to obtain extracts with the highest capacity to reduce micellar cholesterol solubility were the highest temperature (80 °C) and pressure (25 MPa) and the lowest X_CO2_ (0.15). Under these conditions, the predicted RMCS capacity was 74.5%. The conditions enabling the highest extraction yield showed the lowest RMCS capacity, and the conditions enabling the highest RMCS capacity yielded the lowest extraction yield. This can be explained by the fact that the extraction with the highest extraction yields was likely less selective, resulting in the extraction of other compounds that did not affect the RMCS. Moreover, the extract showing the highest RMCS capacity was obtained, above the critical pressure of the mixture, while the extract showing the lowest capacity was obtained, below the critical pressure of the mixture (see ESM Fig. S1). However, the solvent mixture was in a liquid state in all the experiments performed; thus, it is expected that pressure has no influence on the solvent characteristics. Nevertheless, X_CO2_ and temperature might have influence on the solvent behavior in terms of density and solubility. At low temperatures, the density of the mixture increases at higher CO_2_ content, hence increasing solvation capacity and solubility of the compounds [35]. This fact could lead to a poor selectivity in the extraction process since any compound can be solubilized in the solvent. That is why higher yields were obtained under these extraction conditions (see Table 2 and Fig. 2a). On the other hand, at high temperature and lower CO_2_ content, the density of the solvent mixture as well as its solvation capacity decreases [35]. In this sense, more selective extraction processes were performed getting higher RMCS capacities (see Table 2 and Fig. 2b).

In order to identify the compounds responsible for the cholesterol-lowering capacity, the extracts exerting the highest (extract obtained in experiment 10) and the lowest (extract obtained in experiment 1) RMCS capacity were next analyzed by GC–MS.

### GC–MS characterization of CXL extracts from olive seeds

**Fig. 3:**
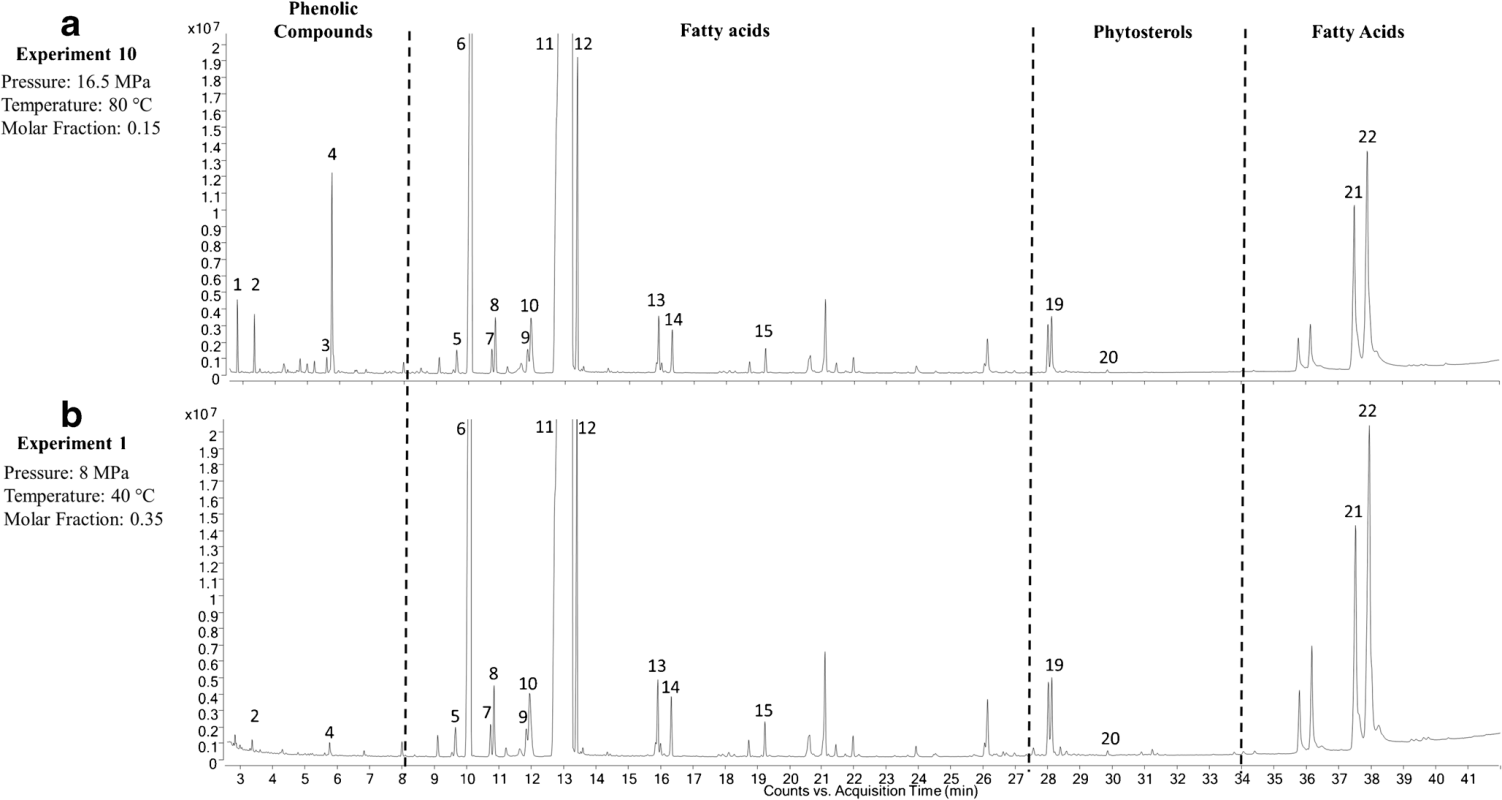
GC–MS chromatograms corresponding to the most active extract (experiment 10) (**a**) and the least active extract (experiment 1) (**b**)

A GC–MS method was set up based on the previous method of the research group [31]. The temperature ramp was modified since there were no peaks from 40 to 150 °C. Moreover, temperature was increased until 350 °C to assure that all compounds were eluted. Figure 3 shows the GC–MS chromatograms corresponding to the extract exerting the highest RMCS capacity, experiment 10 (Fig. 3a), and to the extract exerting the lowest capacity (and higher extraction yield (% *w*/*w*), experiment 1 (Fig. 3b). Additionally, the identified compounds in both extracts with their relative area contribution (expressed as percentage of normalized areas) and the calculated RIs are summarized in Table 4. The separated compounds were identified comparing both spectral data and RIs with the theoretical ones. When available, compounds were identified by comparison with commercial standards. As observed, 23 compounds were identified which could be divided mainly into three groups: phenolic compounds, phytosterols, and free fatty acids (Fig. 3 and Table 4).

**Table 4:** Compounds identified in the extracts showing the highest and the lowest RMCS capacity by GC–MS

Peak	RT	RI	Compound	Formula	Area % (experiment 10)	Area % (experiment 1)
1	2.9	< 1684.4	*cis*-4-Trimethylsilyloxy-cyclohexyl(trimethylsilyl)carboxylate)	C_13_H_28_O_2_Si_2_	0.41	–
2	3.4	< 1684.4	Tyrosol-TMS (*)	C_14_H_26_O_2_Si_2_	0.37	0.10
3	5.7	1684	*p*-Coumaric acid, TMS	C_15_H_26_O_3_Si_2_	0.14	–
4	5.9	1690	Hydroxityrosol-TMS (*)	C_17_H_34_O_3_Si_3_	1.70	0.10
5	9.8	2016	(Palmitoleic acid, ω-7)-TMS	C_19_H_38_O_2_Si	0.23	0.22
6	10.3	2029	Palmitic acid-TMS	C_19_H_40_O_2_Si	10.69	11.06
7	10.9	2047	9,12-Octadecadienoic acid (Z,Z)-,methyl ester	C_19_H_34_O_2_	0.19	0.19
8	11.0	1732	5-Octadecenoic acid, methyl ester	C_19_H_36_O_2_	0.42	0.42
9	12.0	2080	Linoleic acid ethyl ester	C_20_H_36_O_2_	0.25	0.23
10	12.1	2083	Ethyl linoleate / Ethyl oleate	C_20_H_38_O_2_Si	0.74	0.78
12	13.6	2233	Stearic acid-TMS	C_21_H_44_O_2_Si	2.41	2.46
13	16.1	2413	C(23:1)-TMS	C_23_H_46_O_2_Si	0.48	0.47
14	16.5	2427	Arachidic acid-TMS	C_23_H_48_O_2_Si	0.39	0.38
15	19.4	2626	Behenic acid-TMS	C_25_H_52_O_2_Si	0.23	0.22
16	20.8	2675	1-Monooleoylglycerol-TMS	C_27_H_56_O_4_Si_2_	0.56	0.51
17	21.2	2692	1-Monooleoylglycerol-TMS	C_27_H_56_O_4_Si_2_	0.59	0.58
18	22.2	2826	Pentacosanoic acid, trimethylsilyl ester	C_28_H_58_O_2_Si	0.16	0.14
19	28.3	3272	β-sitosterol-TMS (*)	C_32_H_58_OSi	0.51	0.83
20	29.4	3423	1-Monooleoylglycerol-TMS	C_27_H_56_O_4_Si_2_	0.21	0.16
21	37.8	> 3430	Lanosterol	C_30_H_50_O	4.26	4.13
22	38.2	> 3430	Triolein	C_57_H_104_O_6_	3.49	4.88

(*) Available standards

Both chromatograms showed similar profiles and main differences were observed in the areas of phenolic compounds that eluted at short retention times (from 2.5 to 6 min). Three phenolic compounds were identified in the olive seed extract with the highest RMCS (experiment 10): 4-(2-hydroxy-ethyl)phenol (tyrosol, peak 2), 4-hydroxycinnamic acid (*p*-coumaric acid, peak 3), and 4-(2-hydroxyethyl)benzene-1,2-diol (hydroxytyrosol, peak 4). They are the main phenolic compounds found in olive products and olive by-products. According to their chemical structure, phenolic compounds are usually found in the water-soluble parts of olive and its by-products, but there is a small part that could also be found in the oil [36]. Among their biological properties and their health benefits are the reduction of the blood cholesterol levels and the prevention of age-related processes, chronic inflammatory disorders, some cancer diseases, and other diseases related to metabolic syndrome [11]. However, only tyrosol was found at a low intensity in the extract with the lowest in vitro RMCS (experiment 1). The difference in the phenolic abundances between both extracts (experiments 1 and 10) suggested that phenolic compounds were significant contributors to the in vitro cholesterol-lowering capacity.

HSP values were calculated for both experiments (1 and 10) in order to observe what parameters have affected the extraction of cholesterol-lowering compounds. HSP values in the mixture of CO_2_ and ethyl acetate were calculated with Eqs. (1), (2), (3), and (4). HSP for experiment 1 were *δ*_D_ = 11.0, *δ*_P_ = 4.3, *δ*_H_ = 6.6, and *δ*_Total_ = 13.5; and for experiment 10, HSP were *δ*_D_ = 12.9, *δ*_P_ = 4.8, *δ*_H_ = 6.5, and *δ*_Total_ = 15.2. The main differences were in *δ*_D_ and *δ*_P_ that they affect Van der Waals forces and polar interaction, respectively. Therefore, in experiment 10, the polar interaction and Van der Waals forces were higher than in experiment 1 favoring the extraction of phenolic compounds.

Other groups of compounds found in the olive seed extracts were free fatty acids (FFA). FFA fraction eluted in the region from 8 to 27 min and from 34 to 40 min. No difference was observed in the region from 8 to 27 min when comparing both extracts suggesting that these compounds were not mainly responsible for the observed cholesterol-lowering capacity. Main FA in the extracts was oleic acid (peak 11), representing around 65% of total peak area in both extracts. Oleic acid is the most important monounsaturated FFA in the olive and can be present in a wide range of concentrations, from 47 to 77% (*w*/*w*) of oil matter [37, 38]. The intake of unsaturated FA such as oleic acid is related to health benefits due to its capacity to reduce LDL-cholesterol in the blood [39].

Additionally, the plant sterol β-sitosterol was also identified in both extracts (tr = 28.3 min, peak 19) (see Table 4). β-Sitosterol is the most abundant plant sterol found in the vegetable kingdom. Phytosterols are compounds very similar to cholesterol structure. They are extracted from plant oil matrices and are insoluble in water. The interest in phytosterols is based on their documented cholesterol-lowering effects, anti-atherogenicity effect, anticancer properties, and antioxidant and anti-inflammatory activities [40]. As can be seen in Fig. 3 and Table 4, the normalized areas of β-sitosterol in extracts 10 and 1 were around 0.5–0.8%, being slightly higher in the extract with the lowest RMCS capacity (extract of experiment 1).

### Effect of the experimental conditions on the extraction of phenolic compounds, phytosterols, and free fatty acids

In order to predict the experimental conditions enabling the highest extraction of phenolic compounds, FFA and β-sitosterol, the total peak area of every group of compounds obtained in the 15 experiments established by the experimental design was calculated and data were included in Table 2. Phenolic compounds and phytosterol contents were linearly correlated with temperature, pressure, and X_CO2_, while no correlation was observed for the FFA content. Indeed, multiple linear regression enabled to describe more than 81% of the variability of phytosterol and phenolic compound contents. Moreover, ANOVA confirmed that the established regression model was correct for these two responses. Nevertheless, there was not any significant difference among the FFA contents obtained at the tested conditions, which means that these compounds do not contribute to the RMCS capacity observed in the extracts.

Furthermore, Fig. 2c, d shows, as contour plots, the influence of extraction conditions on the phenolic compounds and phytosterol contents. At the lowest concentration of CO_2_, phenolic compound content was positively correlated with temperature, while pressure did not have any effect (see Fig. 2c). This could be because the solubility of phenolic compounds increases when increasing the temperature favoring their extraction [41]. Moreover, ethyl acetate presents a higher dielectric constant than CO_2_, and thus, the dielectric constant of the solvent at the lowest X_CO2_ is greater than at higher X_CO2_ favoring the extraction of phenolic compounds. Thus, the best conditions to extract phenolic compounds were a pressure of 25 MPa, a temperature of 80 °C, and a X_CO2_ of 0.15. As can be seen, higher pressures might increase the extraction of phenolic compounds, but the pressure effect was not significant (*p* ≥ 0.05) (Table 3). This fact was also observed by Prasad et al. in the extraction of phenolic compounds from longan fruit pericarp, for which more than 20 MPa was needed to extract the highest concentration of phenolic compounds [42]. High pressures enable to increase mass transfer since the solvent can disrupt cell walls and hydrophobic bonds in the cell membranes, which lead to a high permeability [43]. Surprisingly, these conditions were identical to those predicting the highest RMCS capacity. In fact, contour plots for phenolic compounds and RMCS were very similar (Fig. 2b, c).

Unlike phenolic compounds, β-sitosterol content was positively correlated with X_CO2_ and pressure (Fig. 2d). This behavior could be expected since at increasing CO_2_ concentration in the solvent, the relative permittivity of the mixture decreases and the interactions between nonpolar compounds and the solvent are favored. Optimal extraction conditions to recover β-sitosterol were a pressure of 25 MPa, a temperature of 62 °C, and a X_CO2_ of 0.55. Similar to phenolic compounds, the extraction of β-sitosterol was favored at high pressures. The extract with the highest β-sitosterol content (experiment 8) also showed a high RMCS capacity (around 62%) (Table 2). Moreover, the extract obtained in experiment 1, which showed a low RMCS capacity, also presented the lowest content of both phenolic compounds and β-sitosterol, while the extract from experiment 10, that showed a high RMCS capacity, presented the highest phenolic content and also a high β-sitosterol content. These results suggested that phenolic compounds were the main contributor to RMCS capacity and that a suitable balance between phenolic compounds and β-sitosterol may affect greatly the RMCS capacity.

## Conclusions

The present work proposes a “green” methodology to extract highly cholesterol-lowering compounds from a food by-product involving the use of CO_2_-expanded bio-based solvents. HSP enabled to predict that ethyl acetate was the most suitable bio-based solvent for the simultaneous extraction of cholesterol-lowering compounds of different polarities from the olive seed. The use of the experimental Box–Behnken design enabled to reduce the number of experiments to 15 to study the influence of temperature, pressure, and X_CO2_ on the extraction. Optimal extraction conditions to obtain the highest RMCS capacity were obtained at a pressure of 25 MPa, a temperature of 80 °C, and a X_CO2_ of 0.15. The analysis of extracts showing the highest and the lowest RMCS capacity by GC–MS enabled to observe that β-sitosterol and, especially, phenolic compounds were the main contributors to the cholesterol-lowering capacity observed in seed extracts. This eco-friendly strategy enables the exploitation of a sustainable source of phenolic compounds and β-sitosterol that have demonstrated to have high capacity to reduce cholesterol solubility and that could be of great interest to produce functional foods and nutraceuticals.

## Electronic supplementary material

ESM 1 (PDF 271 kb)
