# Supplementary material for: A sustainable approach for the extraction of cholesterol-lowering compounds from an olive by-product based on CO2-expanded ethyl acetate
Source: Anal Bioanal Chem. 2019 Jul 6;411(22):5885–96. doi: 10.1007/s00216-019-01970-4 (PMC6704084; doi:10.1007/s00216-019-01970-4)
Supplement: Supplementary file 1 — (PDF 271 kb) [file 216_2019_1970_MOESM1_ESM.pdf]

**Analytical and Bioanalytical Chemistry**

**Electronic Supplementary Material**

# A sustainable approach for the extraction of cholesterol-lowering compounds from an olive by-product based on $CO_2$-expanded ethyl acetate

Romy Vásquez-Villanueva, Merichel Plaza, María Concepción García, Charlotta Turner, María Luisa Marina

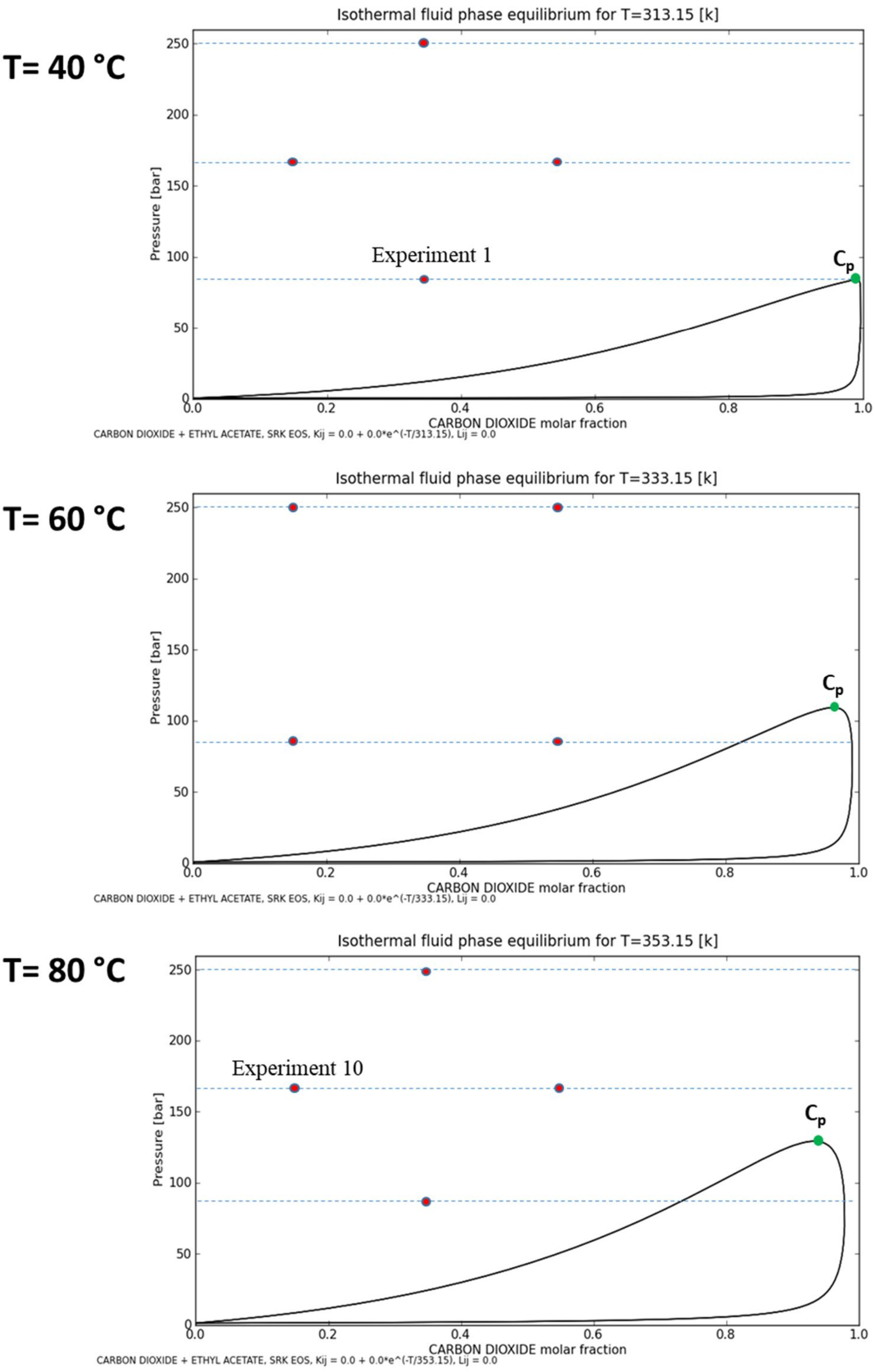


**Fig. S1** Isotherms at 40, 60 and 80 ºC for $CO_2$ and ethyl acetate binary system estimated by the software GPEC (http://phasety.com/?lang=en). The green dots represent the critical point (Cp) and the red dots show the extractions conditions employed on the optimization of hypocholesterolemic compounds recovery from olive seeds by $CO_2$ expanded ethyl acetate. The experiments 10 and 1 displayed the extracts with the highest and the lowest hypocholesterolemic activity, respectively
